# Epidemiological factors associated with human cystic echinococcosis: a semi-structured questionnaire from a large population-based ultrasound cross-sectional study in eastern Europe and Turkey

**DOI:** 10.1186/s13071-019-3634-1

**Published:** 2019-07-29

**Authors:** Francesca Tamarozzi, Okan Akhan, Carmen Michaela Cretu, Kamenna Vutova, Massimo Fabiani, Serra Orsten, Patrizio Pezzotti, Gabriela Loredana Popa, Valeri Velev, Mar Siles-Lucas, Enrico Brunetti, Adriano Casulli

**Affiliations:** 1 WHO Collaborating Centre for the Epidemiology, Detection and Control of Cystic and Alveolar Echinococcosis (in Animals and Humans), Department of Infectious Diseases, Istituto Superiore di Sanità, Rome, Italy; 2 WHO Collaborating Centre for Clinical Management of Cystic Echinococcosis, Pavia, Italy; 3 Department of Radiology, Faculty of Medicine, Hacettepe University, Ankara, Turkey; 4 C. Davila University of Medicine and Pharmacy, Colentina Clinical Hospital, Bucharest, Romania; 5 Specialised Hospital of Infectious and Parasitic Diseases “Prof. Ivan Kirov”, Department of Infectious, Parasitic and Tropical Diseases, Medical University, Sofia, Bulgaria; 6 Unit of Epidemiology, Biostatistics and Mathematical Modelling, Department of Infectious Diseases, Istituto Superiore di Sanità, Rome, Italy; 7 School of Health Services, Hacettepe University, Ankara, Turkey; 8 Instituto de Recursos Naturales y Agrobiología de Salamanca, CSIC, Salamanca, Spain; 9 Department of Clinical Surgical Diagnostic and Pediatric Sciences, University of Pavia, Pavia, Italy; 10 Division of Tropical and Infectious Diseases, San Matteo Hospital Foundation, Pavia, Italy; 11 European Reference Laboratory for Parasites, Department of Infectious Diseases, Istituto Superiore di Sanità, Rome, Italy

**Keywords:** Cystic echinococcosis, Epidemiology of human infection, Potential risk factors, Semi-structured questionnaires, Eastern Europe, Romania, Bulgaria, Turkey

## Abstract

**Background:**

Cystic echinococcosis (CE) is a neglected parasitic zoonosis prioritized by the WHO for control. Several studies have investigated potential risk factors for CE through questionnaires, mostly carried out on small samples, providing contrasting results. We present the analysis of risk factor questionnaires administered to participants to a large CE prevalence study conducted in Bulgaria, Romania and Turkey.

**Methods:**

A semi-structured questionnaire was administered to 24,687 people from rural Bulgaria, Romania and Turkey. CE cases were defined as individuals with abdominal CE cysts detected by ultrasound. Variables associated with CE at *P* < 0.20 in bivariate analysis were included into a multivariable logistic model, with a random effect to account for clustering at village level. Adjusted odds ratios (AOR) with 95% CI were used to describe the strength of associations. Data were weighted to reflect the relative distribution of the rural population in the study area by country, age group and sex.

**Results:**

Valid records from 22,027 people were analyzed. According to the main occupation in the past 20 years, “housewife” (AOR: 3.11; 95% CI: 1.51–6.41) and “retired” (AOR: 2.88; 95% CI: 1.09–7.65) showed significantly higher odds of being infected compared to non-agricultural workers. “Having relatives with CE” (AOR: 4.18; 95% CI: 1.77–9.88) was also associated with higher odds of infection. Interestingly, dog-related and food/water-related factors were not associated with infection.

**Conclusions:**

Our results point toward infection being acquired in a “domestic” rural environment and support the view that CE should be considered more a “soil-transmitted” than a “food-borne” infection. This result helps delineating the dynamics of infection transmission and has practical implications in the design of specific studies to shed light on actual sources of infection and inform control campaigns.

**Electronic supplementary material:**

The online version of this article (10.1186/s13071-019-3634-1) contains supplementary material, which is available to authorized users.

## Background

Cystic echinococcosis (CE) is a parasitic zoonotic disease caused by infection with the larval stage (metacestode) of the tapeworm *Echinococcus granulosus* *sensu lato* species complex. Its natural life-cycle develops between canids (definitive hosts harbouring the adult stage in the intestine) and ungulates (intermediate hosts developing the larval stage in internal organs), in a predator-prey transmission pathway. The majority of human cases are documented in rural areas where livestock breeding is practised, consistent with a life-cycle mainly involving sheep and dogs [1, 2]. CE has remarkable health and socio-economic consequences for the rural populations affected [3, 4]. Current global estimates indicate a prevalence of 1–3 million cases of human CE, with a burden of 1–3.6 million disability adjusted life years and over 2 billion US$ costs accounting for human treatment and livestock production losses [4, 5]. In 2014, a joint FAO/WHO expert meeting ranked CE as the third most important food-borne parasitic disease at the global level [6]. Furthermore, in 2018 EFSA published a scientific opinion on public health risks associated with food-borne parasites, highlighting CE as “of the highest relevance in Europe” [7]. The WHO indicates CE as a zoonosis prioritized for control actions, including in Europe [8, 9].

Humans represent an accidental “dead-end” intermediate host for the metacestode of *E. granulosus*, thus they do not contribute to the perpetuation of the parasite’s life-cycle. In the presence of ongoing transmission between natural animal hosts, only primary prevention measures may reduce sustainably, in the long term, the burden of CE in humans. Transmission among animals, in turn, may be controlled though implementation of abattoir surveillance and safe disposal of offal, culling of aged sheep, periodic deworming of dogs with praziquantel and vaccination of sheep [10]. Hygiene education is one of the strategies included in CE control campaigns; however, on its own, this intervention did not impact significantly on transmission rate to humans [10].

Humans acquire infection through oral uptake of infective *E. granulosus* eggs; however, there is a great uncertainty on the actual source attribution and precise risk factors for infection. “Ingestion of contaminated food and water”, together with “direct contact/playing with dogs” are classically mentioned as the sources of human infection and are biologically plausible potential risk factors. However, actual data on contamination of and relative attribution from such sources are extremely scant and uncertain [7, 11, 12]. Furthermore, Chaabane-Banaoues et al. [13] found that degree of environmental contamination by *E. granulosus*-positive dog faeces did not necessarily correlate with human prevalence of CE, highlighting that multiple ecological factors, likely varying from area to area, and involving human behaviour and hygiene habits, are at the basis of human transmission.

Knowing the dynamics of infection transmission to humans in endemic areas may allow optimizing and increasing the effectiveness of interventions aiming at the reduction of eggs ingestion by humans. Several studies investigated the potential risk factors associated with human CE through questionnaires administered in hospital-based case-control and field-based cross-sectional studies, mostly providing contrasting results. Recently a systematic review and meta-analysis by Possenti et al. [14] aiming to summarize available data on statistically relevant potential risk factors, indicated that “living in endemic areas” and “dog ownership” seem to be the most significant potential risk factors for acquiring CE, consistently resulting from both case-control and cross-sectional studies. Conversely, “dog contact” had a weak and non-significant association [14]. The same systematic review also found that factors related to habits involved in the perpetuation of the parasite life-cycle (type of slaughtering, feeding dogs with raw viscera) were associated with increased risk of infection with variable statistical significance, while food- and water-borne pathways of transmission did not appear to impact significantly on the risk of humans acquiring CE [14]. Environmental contamination was also identified as the main factor associated with CE in more recent cross-sectional surveys carried out in endemic areas of Morocco [15] and Peru [16]. Factors associated with both parasite life-cycle perpetuation and transmission through food or water were, on the contrary, reported as significantly associated with household risk of human CE in a recent Chinese study [17].

Previous questionnaire-based studies investigating potential risk factors associated with human CE generally tested samples of limited sizes and differed greatly one from the other for what concerns data collected in the interviews [14]. Furthermore, only about half of the field-based cross-sectional studies investigating risk factors through questionnaires used imaging as the diagnostic methodology for case definition, therefore confirming actual CE [14]. In 2014–2015, we carried out a large research-based field survey on human CE in the context of the project “Human cystic Echinococcosis ReseArch in CentraL and Eastern Societies” (HERACLES) [18] funded by the European Commission in 2013. Using abdominal ultrasound, we examined 24,687 people from rural areas of Romania, Bulgaria and Turkey, estimating that 151,000 people may have abdominal CE in the rural areas on these countries, a third of them potentially requiring treatment [19]. Here we present the analysis of risk factors questionnaires administered to participants during the HERACLES ultrasound population-based surveys.

## Methods

### Ultrasound surveys

A detailed description of the ultrasound surveys has been published [19]. Briefly, abdominal ultrasound screening sessions were performed in 2014–2015 on 24,687 people in 50 villages of Bulgaria, Romania and Turkey, in areas of mid-range endemicity for human CE. The primary aim of the study was to estimate the prevalence of abdominal CE, cyst stage distribution and number of infected individuals in the rural populations of these countries. The screenings were carried out on the convenience sample of all volunteers living in the targeted endemic provinces who presented to the sessions in each village and signed the informed consent form. In all countries, a common protocol for diagnosis and clinical management of CE based on the WHO Informal Working Group on Echinococcosis (WHO-IWGE) Expert Consensus on clinical management of echinococcosis [20] was applied. Health education on CE was provided during the pre-screening project-advertising activities and by the project team during the surveys with the aid of paper-based and audio-visual supports.

### Questionnaires

A semi-structured paper-based questionnaire (Additional file 1) written in Bulgarian, Romanian and Turkish was administered by the survey staff individually to each participant. Children, where appropriate, were helped to answer by parents/guardians. The questionnaire was administered after signing the informed consent form and before the ultrasound exam. The questionnaire included demographic, occupational and schooling-related questions, as well as questions concerning knowledge about existence of human CE and occurrence of cases in the family, dog and livestock-related practices, and food- and drinking water-related habits. After the field surveys, data were transferred to an electronic database (Microsoft Excel), and manually curated before analysis. The complete list of variables and their categorization for analysis is presented in Additional file 2: Table S1.

### Case definition

A detailed description of classification of patients and CE cysts has been published [19]. In brief, infection with CE was based on ultrasound imaging, evaluated by two sonographers during the screening and confirmed by re-evaluation of each lesion through images and video files before data analysis. Cysts were identified and staged based on the visualization of pathognomonic signs of CE aetiology, according to the WHO-IWGE Expert Consensus [20]; more stringent conditions were, however, applied to unilocular cysts, which where ascribed to parasitic aetiology only if a double wall was clearly visible. Lesions suspect of CE, including cystic lesions (CL), were investigated as per protocol to define the nature of the lesion [19].

Due to logistic constraints, only patients visiting the project’s referral hospitals for treatment of CE cysts received a chest X-ray for the detection of possible lung CE; none were positive [19]. “CE cases” were defined as all individuals with abdominal CE cysts detected on ultrasound, independently of whether they reported having received previous treatment for CE.

### Statistical analysis

We excluded from the analysis questionnaires from 128 (0.5%) individuals who had no CE cysts detected by imaging, but self-reported treatment for CE in the past, as it was not possible to confirm their infection through clinical documentation. Moreover, we excluded questionnaires with incomplete information (*n* = 2426; 9.8%) or unresolvable incongruences (*n* = 106; 0.4%), leaving complete records from 22,027 (89.3%) participants available for the analysis. Individuals (*n* = 38) with suspect lesions, the aetiology of which could not be ascertained, were considered as CE-negative. We described the socio-demographic characteristics and risk profile of the study sample population through counts and percentages.

The prevalence of CE was estimated using sampling weights to reflect the relative distribution of the rural population in the study area by country, age group and sex, as derived from official population statistics [19]. Prevalence estimates were presented with 95% confidence intervals (CI), calculated using the Taylor linearization method to account for the increased variance due to the sampling design. The association between the presence of CE and each potential risk factor was evaluated using a Chi-square test on the whole sample of 22,027 questionnaires, based on the geographical and ecological contiguity of the entire investigated area. All variables associated with CE at *P* < 0.20 in bivariate analysis were included into a multivariable logistic model together with a random effect to account for clustering at village level. Regardless of its association with CE in bivariate analysis, we excluded “current occupation” from the multivariable analysis to prevent collinearity problems due to its strong association with the variable “prevalent occupation in the past 20 years” (we assumed the latter as more appropriate to evaluate the occupation-related risk for an infection that was likely acquired years previously). We scaled sampling weights according to the actual clusters’ size before running the multilevel model [21]. The adjusted odds ratio (AOR) with 95% CI were used to describe the strength of the associations. The interactions between each risk factor and country were assessed through the Wald test. Country-specific multivariable models were also computed. Finally, we used the intraclass correlation coefficient (ICC) to estimate the proportion of the residual variability attributable to the village-related context [22]. Statistical significance was set at *P* < 0.05. The analysis was performed using Stata/MP v.14.2 (StataCorp LP, College Station, TX, USA).

## Results

Of 22,027 analysed questionnaires, 13,957 (63.4%) referred to females and 8070 (36.6%) to males; 105 people (0.51%, 95% CI: 0.24–1.07%) had abdominal CE during the ultrasound screenings [71 females (0.63%, 95% CI: 0.25–1.55%) and 34 males (0.39%, 95% CI: 0.23–0.67%)]. The results of the descriptive and bivariate analysis performed on the whole sample are presented in Additional file 2: Table S1. Ten of the 23 analysed variables were associated with CE at *P* < 0.20 and included into the multivariable logistic model.

**Fig. 1:**
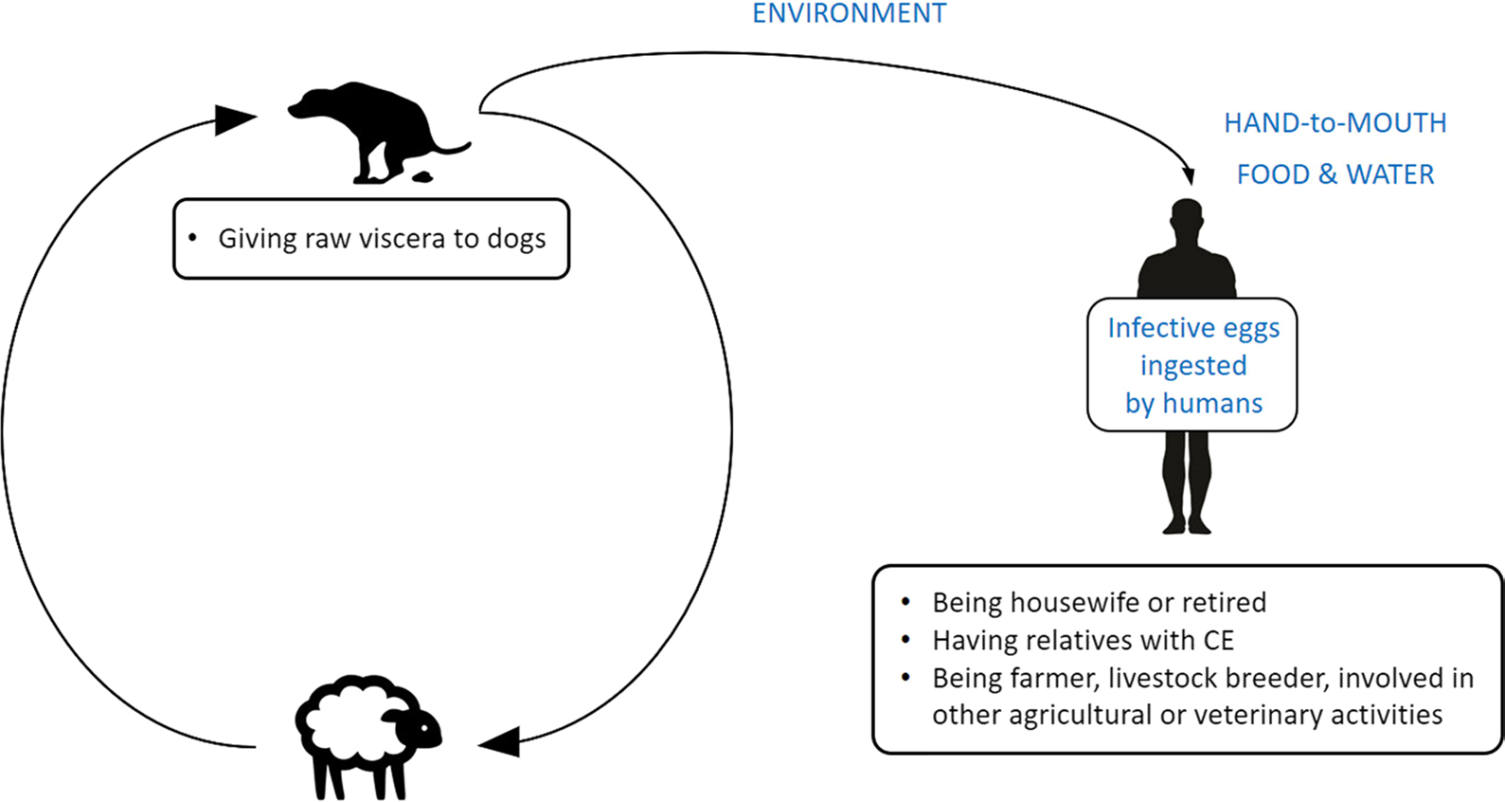
Epidemiological factors associated with increased odds of human cystic echinococcosis (CE). Schematic representation of the *E. granulosus* life-cycle, pathways of transmission to humans (in blue), and potential risk factors associated with increased odds of human infection identified in our study (bullet points)

The results of the multivariable analysis are presented in Table 1 and graphically depicted in Fig. 1. In relation to the main occupation in the past 20 years, housewives (AOR: 3.11; 95% CI: 1.51–6.41; *P* = 0.002) and retired persons (AOR: 2.88; 95% CI: 1.09–7.65; *P* = 0.033) showed an increased odds of infection compared to non-agricultural or office/service workers. Having had relatives with CE was positively associated with having CE (AOR: 4.18; 95% CI: 1.77–9.88; *P* = 0.001), while individuals with university or higher level of education showed a significantly reduced odds of infection compared to those without any formal education (AOR: 0.11; 95% CI: 0.01–0.88; *P* = 0.038). Other factors were associated with an increased odds of CE but results were only borderline significant (*P* < 0.1). These were: “Farmer\livestock breeder\other agricultural or veterinary activities as the main occupation in the past 20 years” (AOR: 2.49; 95% CI: 0.93–6.66; *P* = 0.068) and “Giving raw viscera to dogs” (AOR: 1.50: 95% CI: 0.95–2.38; *P* = 0.080). “Drinking commercial water” was associated with a reduced odds of CE with borderline significance (AOR: 0.65; 95% CI: 0.40–1.04; *P* = 0.071).

**Table 1:** Results of the multi-level logistic regression model, including village as random effect

Variable	Adjusted OR	95% CI^a^	*P*-value^a^
Sex			
Female	1		
Male	0.98	0.64–1.5	0.930
Age^b^	1.05	0.89–1.23	0.582
Lived in areas with high density of dogs and sheep in the past 20 years			
No	1		
Yes	1.94	0.84–4.47	0.118
Main occupation in the past 20 years*			
Non-agricultural activities or office/service employee	1		
Housewife	3.11	1.51–6.41	0.002
Farmer/livestock breeder/other agricultural/veterinary activities	2.49	0.93–6.66	0.068
Students and children < 5 years of age	1.31	0.61–2.85	0.487
Retired	2.88	1.09–7.65	0.033
Unemployed	2.09	0.51–8.58	0.309
Agricultural activities in the past 20 years			
No	1		
Yes	1.47	0.64–3.36	0.366
Education			
None	1		
Primary	1.05	0.55–2.00	0.893
Secondary/high school	1.15	0.60–2.18	0.678
University/postgraduate	0.11	0.01–0.88	0.038
Knowledge of human CE existence			
No	1		
Yes	1.78	0.69–4.58	0.235
Known presence of relatives with CE			
No	1		
Yes	4.18	1.77–9.88	0.001
Raw viscera given to dogs			
No	1		
Yes	1.50	0.95–2.38	0.080
Drink commercial water*			
No	1		
Yes	0.65	0.40–1.04	0.071

^a^Accounting for clustering at village level

^b^Adjusted OR per linear 10-years increase in age

* Statistically significant interaction with country (Wald test, *P* < 0.05)

Interestingly, owned dog-related factors (owning dogs and length of dog ownership, reason for keeping dogs, allowing dogs to roam or enter the house, antiparasitic treatment of dogs) were not found associated with odds of human infection (Additional file 2: Table S1; *P*-values of 0.289–0.999). Additionally, the food-related variable “eating unwashed vegetables” (Additional file 2: Table S1) was not associated with odds of CE (*P* = 0.823).

The estimate of the intraclass correlation coefficient (ICC = 16.6%; 95% CI: 7.2–33.7%) indicates that almost one fifth of the residual variability not explained by the individual-level variables included into the multivariable model was likely due to village-related contextual factors.

A significant interaction with country was found only for “Main occupation in the past 20 years” and “Drinking commercial water” (Wald test, *P* < 0.05). The results of the country-specific multivariable models are presented in Additional file 3: Table S2. In general, these were consistent with results from the analysis of the whole sample. However, in Bulgaria, a statistically significant increase in odds of CE was observed for “Student or children < 5 years as the main occupation in the past 20 years” (AOR: 3.04; 95% CI: 1.10–8.32; *P* = 0.032) and individuals with “Primary-level” highest education (AOR: 2.98; 95% CI: 1.15–7.69; *P* = 0.024), while these associations, although not statistically significant, appeared reversed in Romania and Turkey. Moreover, a significantly increased risk of being infected was observed in individuals reporting past agricultural activities in Turkey (AOR: 2.91; 95% CI: 1.50–5.67; *P* = 0.002), but not in those living in Romania and Bulgaria. All the other investigated associations were found to be not statistically significant or having the same direction in all countries.

## Discussion

The WHO advocates control of CE [8]. Reference control strategies include “health education”; however, the content and target population of such educational intervention(s) varied between campaigns and, overall, did not appear to have significantly affected the transmission of CE to humans [10]. Knowing more precisely the human infection risk factors in endemic areas may allow hygiene-based educational interventions that aim to reduce egg ingestion by humans to be optimized and modelled. However, this is particularly difficult due to the absence of symptoms of “acute” human infection and the unknown, likely months to years-long, interval between infection and diagnosis. Multiple potential habits/sources may result in human ingestion of infective parasite eggs. However, so far very few experimental data are available on the actual contamination of different materials by *E. granulosus* eggs [12], and the analyses of questionnaires investigating potential risk factors gave contrasting results [14]. Our questionnaire-based study, carried out in the context of a large research-based cross-sectional prevalence study on human CE [19], applying stringent case definition, may help to better frame the general characteristics of risk factors for human infection.

In our study, factors related to overall lifestyle habits/conditions (e.g. occupation in the past 20 years) were significantly associated with odds of infection. These factors represent and encompass different behaviours and ways of acting that may have allowed the person to be exposed to the infection over time. In particular, variables related to life in the community, such as household-related (housewife, retired) and agricultural-related occupations, were associated with an increased odds of infection; this points toward infection being acquired, in general, in a “domestic” rural environment where the parasite circulates. The increased risk of infection associated with having relatives with CE may also derive from living in a context where human CE is common, and therefore where its transmission cycle is perpetuated. A similar explanation may apply to the trend toward an increased risk of CE associated with having some knowledge of the existence of human CE. On the other hand, drinking commercial water, as well as having a high education, were associated with a reduced odds of infection. These variables may be interpreted as factors indicating high socio-economic status and therefore possibly reduced opportunities of contact with egg-contaminated matrices. The lack of association with sex and age could be explained by the common exposure, in rural areas, of both sexes and at all ages. However, although not statistically significant, an increase in infection prevalence with age can be observed in our data set, as expected for a chronic infection.

The parasite transmission cycle is perpetuated by the habit of giving raw viscera to dogs and this was individuated as a risk factor of borderline significance in our analysis. This behaviour induces dog infection and in turn environmental contamination through shedding of infected faeces. The fact that another habit potentially favouring the transmission of *E. granulosus* to dogs, i.e. home slaughtering of livestock, was not associated with increased odds of infection may be due to the common habit of obtaining viscera to feed dogs even if the household itself did not own livestock and/or did not carry out informal slaughter, as also occurs in other geographical areas [15]. Interestingly, owned dog-related factors (owning dogs and length of dog ownership, reason for keeping dogs, allowing dogs to roam or enter the house, antiparasitic treatment of dogs) were not found associated with odds of human infection. This may derive from the dog husbandry habits in the investigated areas, which may allow environmental contamination with parasite eggs by infected dog faeces in different areas of the community, independently of the dog ownership by the interviewed person. Additionally, a variable meaning of “owning” a dog in different areas, as noted in previous studies, may have influenced this result [15], as well as the possible change over time of these dog-related behaviours. Indeed, in our study, precise habits that may have changed over time or may have been put in practice with variable frequency, such as consumption of unwashed vegetables and of potentially unsafe water, were not associated with odds of infection. On the other hand, our results may also suggest that food- and water-borne transmission may not play a major role as an infection source.

Our study has some limitations deserving discussion. First, limitation deriving from recall bias is intrinsic to the study design and the peculiarity of a chronic, often asymptomatic, infection such as CE. Longitudinal studies would be ideal to investigate the causal relationship between potential risk factors and CE. However, they are virtually impossible to conduct on CE, which is a low-incidence chronic infection, patchily distributed in rural underserved poor areas, often asymptomatic for a long time and with no signs of acute infection. Secondly, it is possible the health education information provided before the administration of the questionnaire would have influenced the answer to the question related to knowledge of CE in humans. However, if this was systematically the case, it would have rather resulted in no association between infection and answer to this question, contrary to what we found. Thirdly, it was difficult to ascertain whether participants replied to questions in terms of their “common habits” or “even occasional” behaviours. This was evident from the mismatch observed between related questions such as: “Do you leave dogs free to roam”, reply “No”, and “Reasons to keep dogs”, reply “Hunting” or “Herding”; “What do you feed dogs with”, reply “Only commercial/cooked food”, and “How do you dispose of viscera”, reply “Give raw to dogs”; “Agricultural activities carried out in the past 20 years”, reply “No”, but then reporting agriculture-related current and/or past occupations. Similarly, the lack of details given regarding the degree of washing/rinsing of vegetables before consumption as well as the broad inclusion of “vegetables” in the question, interpretable as both eaten raw and cooked, may have influenced the results related to these factors. These problems could have been reduced, in part, by pre-testing the questionnaire, which was not carried out due to time constraints deriving from the organizational timeline of the ultrasound surveys. Fourthly, another source of bias may have derived from the convenience sampling strategy, as this may have facilitated the specific participation of some categories of people, thus leading to an overrepresentation of some risk factors. Furthermore, voluntary participation could have introduced self-selection bias as described [19]. The fieldwork was organized in such way to include weekends and extended hours during the day, to allow also working people and students to participate. However, bias deriving from this type of sampling cannot be excluded. Another limitation may derive from the possible inclusion of some individuals with CE among the non-infected group. This may have derived from the stringent case definition applied in the survey [19], and the impossibility of performing a chest radiograph to all survey participants for the detection of isolated lung cysts. Finally, it is worth highlighting that the variables included in our questionnaires were not investigating infection transmission behaviours directly, but can be regarded as indirect driving factors related to socio-economic status and general habits.

Notwithstanding these limitations, however, our results are overall in line with those of the systematic review of Possenti et al. [14], and with the recent studies carried out in Peru and Morocco [15, 16], suggesting environmental contamination as the main risk factor for CE transmission, possibly though a “hand-to-mouth” mechanism.

## Conclusions

The results of our study, carried out in the context of a large research-based cross-sectional study conducted on CE [19], support the view that CE may be considered an “environmental-borne” infection, similar to the “classical” soil-transmitted helminthiases, plausibly transmitted through a “hand-to-mouth” mechanism, while food/water-borne transmission may possibly be of secondary importance. In both cases, however, the “community risk” in endemic areas should be highlighted, aside of “individual” risk factors. More country/community-specific and habits-specific questionnaires, as well as experimental studies on parasite contamination of matrices, are needed to shed light on actual sources of infecting eggs and on behaviours at risk for individual infection. However, these framed concepts, supported by our results, help to delineate the general dynamics of infection transmission and have important practical implications for public health policy makers across endemic countries in the design of control campaigns.

## Additional files

**Additional file 1.** Questionnaire in English. Semi-structured paper-based questionnaire (English version).

**Additional file 2.** Bivariate analysis. Results of the bivariate analysis performed on the whole sample.

**Additional file 3.** Country specific multivariable analysis. Results of the multilevel logistic regression model, including village as random effect.

## Data Availability

Data supporting the conclusions of this article are included within the article and its additional files. The datasets analysed during the present study are available from the corresponding author upon reasonable request.

## Abbreviations

AOR: adjusted odds ratio
CE: cystic echinococcosis
CI: confidence interval
CL: cystic lesion
EFSA: European Food Safety Authority
FAO: Food and Agriculture Organization of the United Nations
ICC: interclass correlation coefficient
WHO: World Health Organization
WHO-IWGE: World Health Organization-Informal Working Group on Echinococcosis
