# Supplementary material for: Epidemiological factors associated with human cystic echinococcosis: a semi-structured questionnaire from a large population-based ultrasound cross-sectional study in eastern Europe and Turkey
Source: Parasit Vectors. 2019 Jul 29;12:371. doi: 10.1186/s13071-019-3634-1 (PMC6664724; doi:10.1186/s13071-019-3634-1)
Supplement: Supplementary file 1 — Additional file 1. Questionnaire in English. Semi-structured paper-based questionnaire (English version). [file 13071_2019_3634_MOESM1_ESM.docx]

**PARTICIPANT DATA Code ________________________ Date ___/___/____**

Country Number

Name ___________________________________ Surname _________________________________

Sex M F Date of birth ____/____/____ Place of birth _____________________________

Residence: City ___________________________ Province _________________________________

Postal code ____________________ Street ___________________________________

Since when ____________________ Contact telephone _________________________

Province/Country of longest residence in past 20 years___________________________

Did you live in areas with dogs and sheep in the past 20 years? Y N

Present occupation _____________________ Occupation in past 20 years_____________________

Agricultural activities (in past 20 years)___________________ Years of school attended __________

| 1) Do you know these lesions in the liver of animals? (show picture of CE) Y N  2) Have you ever heard of echinococcosis/ hydatid disease in humans? Y How?________________ N  3) Do you know if any of your relative has/had cystic echinococcosis? Y How many?___________ N |
| --- |
| 4) Do you slaughter animals at home? Y What? _______________ N |
| 4.1) how do you dispose of viscera? Bury/Burn Give to dogs raw Other___________________  cooked |
| 4.2) what time of the year do you slaughter at home? _________________________________________ |
| 5) Do you keep dogs? Y N |
| 5.1) How many?______ For what reason? Pets Guard Herding Other __________________ |
| 5.2) For how many years did you own dogs? _____ years |
| 5.3) Do you leave the dogs free to roam? Yes in garden Yes in fields Yes everywhere No |
| 5.4) Are dogs allowed into the house? Y N |
| 5.5) What do you feed dogs? Commercial food Cooked food Raw viscera |
| 5.6) Do you treat your dogs with praziquantel? Y How often? ___________________ N |
| 6) Do other dogs (not owned) have access to your territory (garden, fields)? Y N Don’t know |
| 7) How do you dispose of dogs faeces in your territory (garden)? ____________________________________ |
| 8) Do you eat unwashed vegetables? Yes from my garden /fields Yes from the market No |
| 9) What source of water do you use? Tap Commercial Well Other___________________ |
| 10) Do your dogs and livestock can share the same source of water in your properties? Y N  11) Do you have contact with foxes? Y Material_____________ Reason_________________ N |

**VARIABLES CATEGORIZATION FOR ANALYSIS**

The analysis of the answers to the question “years of school attended” was carried out in four “Education” categories. Similarly, the question relative to dog’s treatment with praziquantel was analysed in four categories. Occupations were grouped into six categories. The answers to the questions “Do you leave dogs free to roam” and “What do you feed dogs with” were double-checked for incongruences and manually curated based on answers to the related questions “Reasons for keeping dogs” and “How do you dispose of viscera”. Similarly, the answers whether the interviewed carried out agricultural activities in the past 20 years were double-checked for incongruence and manually curated based on current and past occupation. Ways of disposal of viscera from slaughtered animals was analysed irrespective of whether the person carried out home slaughter, as it is common practice in the investigated areas to obtain livestock viscera that are fed to dogs even without owning livestock and/or slaughtering livestock at home. Finally, due to the extreme heterogeneity of the answers provided, we could not analyse the answers to the questions addressing the frequency of antiparasitic treatment of dogs, the recognition of the picture of a CE cyst in the liver of a sheep, the time of the year in which home slaughter was carried-out, the ways of disposal of dogs’ feces, and the access of dogs not owned by the interviewed in his/her household facilities.
