# Supplementary material for: Epidemiological factors associated with human cystic echinococcosis: a semi-structured questionnaire from a large population-based ultrasound cross-sectional study in eastern Europe and Turkey
Source: Parasit Vectors. 2019 Jul 29;12:371. doi: 10.1186/s13071-019-3634-1 (PMC6664724; doi:10.1186/s13071-019-3634-1)
Supplement: Supplementary file 2 — Additional file 2. Bivariate analysis. Results of the bivariate analysis performed on the whole sample. [file 13071_2019_3634_MOESM2_ESM.docx]

**Additional file 2: Table S1. Results of the bivariate analysis performed on the whole sample**. *Calculated by weighting data according to the rural population size by country, sex and age group. ** Accounting for clustering at village level. NC, not calculable.

| **Variable** | **N** | **%** | **N CE cases** | **Prevalence %*** | **95% CI**** | ***P*-value**** |
| --- | --- | --- | --- | --- | --- | --- |
| SEX |  |  |  |  |  | 0.095 |
| Female | 13,957 | 63.4 | 71 | 0.63 | 0.25-1.55 |  |
| Male | 8,070 | 36.6 | 34 | 0.39 | 0.23-0.67 |  |
| AGE GROUP |  |  |  |  |  | 0.002 |
| <20 | 5,139 | 23.3 | 11 | 0.20 | 0.06-0.60 |  |
| 20-29 | 1,650 | 7.5 | 10 | 0.70 | 0.24-2.03 |  |
| 30-39 | 2,892 | 13.1 | 14 | 0.47 | 0.19-1.14 |  |
| 40-49 | 3,461 | 15.7 | 15 | 0.48 | 0.25-0.90 |  |
| 50-59 | 3,539 | 16.1 | 21 | 0.76 | 0.43-1.37 |  |
| ≥60 | 5,346 | 24.3 | 34 | 0.77 | 0.34-1.72 |  |
| LIVED IN AREAS WITH HIGH DENSITY OF DOGS AND SHEEP  IN THE PAST 20 YEARS |  |  |  |  |  | 0.053 |
| No | 5,007 | 22.7 | 11 | 0.16 | 0.08-0.35 |  |
| Yes | 17,020 | 77.3 | 94 | 0.53 | 0.25-1.15 |  |
| CURRENT OCCUPATION |  |  |  |  |  | 0.006 |
| Non-agricultural activities or office/service employee | 5,867 | 26.6 | 21 | 0.44 | 0.26-0.75 |  |
| Housewife | 3,499 | 15.9 | 33 | 1.02 | 0.33-3.14 |  |
| Farmer/livestock breeder/other agricultural/veterinary activities | 2,670 | 12.1 | 12 | 0.55 | 0.26-1.16 |  |
| Students and children <5 years of age | 5,079 | 23.1 | 10 | 0.17 | 0.06-0.47 |  |
| Retired | 3,911 | 17.8 | 23 | 0.66 | 0.39-1.12 |  |
| Unemployed | 1,011 | 4.5 | 6 | 0.27 | 0.09-0.86 |  |
| MAIN OCCUPATION IN THE PAST 20 YEARS^#^ |  |  |  |  |  | <0.001 |
| Non-agricultural activities or office/service employee | 7,634 | 34.7 | 21 | 0.34 | 0.16-0.69 |  |
| Housewife | 2,743 | 12.5 | 31 | 1.24 | 0.40-3.81 |  |
| Farmer/livestock breeder/other agricultural/veterinary activities | 4,466 | 20.3 | 28 | 0.63 | 0.40-0.99 |  |
| Students and children <5 years of age | 5,361 | 24.3 | 12 | 0.17 | 0.07-0.46 |  |
| Retired | 1,088 | 4.9 | 9 | 0.78 | 0.40-1.49 |  |
| Unemployed | 735 | 3.3 | 4 | 0.25 | 0.07-0.93 |  |
| AGRICULTURAL ACTIVITIES IN THE PAST 20 YEARS |  |  |  |  |  | <0.001 |
| No | 10,377 | 47.1 | 31 | 0.31 | 0.15-0.65 |  |
| Yes | 11650 | 52.9 | 74 | 0.67 | 0.30-1.52 |  |
| EDUCATION |  |  |  |  |  | 0.175 |
| None | 2,623 | 11.9 | 17 | 0.77 | 0.20-2.94 |  |
| Primary | 10,017 | 45.5 | 54 | 0.49 | 0.29-0.83 |  |
| Secondary/High school | 7,756 | 35.2 | 33 | 0.52 | 0.22-1.21 |  |
| University/Postgraduate | 1,631 | 7.4 | 1 | 0.01 | 0.00-0.07 |  |
| KNOWLEDGE OF HUMAN CE EXISTENCE |  |  |  |  |  | <0.001 |
| No | 18,854 | 85.6 | 69 | 0.38 | 0.22-0.65 |  |
| Yes | 3,173 | 14.4 | 36 | 1.56 | 0.63-3.82 |  |
| KNOWN PRESENCE OF RELATIVES WITH CE |  |  |  |  |  | <0.001 |
| No | 21,169 | 96.1 | 82 | 0.41 | 0.24-0.69 |  |
| Yes | 858 | 3.9 | 23 | 3.50 | 1.24-9.50 |  |
| SLAUGHTER LIVESTOCK AT HOME |  |  |  |  |  | 0.359 |
| No | 7,857 | 35.7 | 28 | 0.37 | 0.22-0.62 |  |
| Yes | 14,170 | 64.3 | 77 | 0.55 | 0.24-1.26 |  |
| VISCERA DISPOSED BY BURY/BURN |  |  |  |  |  | 0.941 |
| No | 17,564 | 79.7 | 83 | 0.51 | 0.24-1.10 |  |
| Yes | 4,463 | 20.3 | 22 | 0.50 | 0.24-1.07 |  |
| RAW VISCERA GIVEN TO DOGS |  |  |  |  |  | <0.001 |
| No | 16,929 | 76.9 | 63 | 0.37 | 0.19-0.75 |  |
| Yes | 5,098 | 23.1 | 42 | 0.84 | 0.41-1.73 |  |
| COOKED VISCERA GIVEN TO DOGS |  |  |  |  |  | 0.260 |
| No | 16,598 | 75.4 | 86 | 0.56 | 0.25-1.27 |  |
| Yes | 5,429 | 24.6 | 19 | 0.30 | 0.14-0.66 |  |
| OWNING DOGS |  |  |  |  |  | 0.993 |
| No | 11,963 | 54.3 | 56 | 0.51 | 0.24-1.10 |  |
| Yes | 10,064 | 45.7 | 49 | 0.51 | 0.24-1.10 |  |
| REASONS FOR KEEPING DOGS |  |  |  |  |  | 0.681 |
| No dogs | 11,963 | 54.3 | 56 | 0.51 | 0.24-1.10 |  |
| Pets | 748 | 3.4 | 2 | 0.26 | 0.08-0.83 |  |
| Guard | 7,078 | 32.1 | 35 | 0.49 | 0.24-0.98 |  |
| Herding | 2,148 | 9.8 | 12 | 0.83 | 0.24-2.85 |  |
| Hunting | 90 | 0.4 | 0 | 0.00 | NC |  |
| TIME SINCE OWING DOGS |  |  |  |  |  | 0.879 |
| No dogs | 11,963 | 54.3 | 56 | 0.51 | 0.24-1.10 |  |
| < 5 years | 2,085 | 9.5 | 11 | 0.50 | 0.12-2.06 |  |
| 5-10 years | 1,820 | 8.3 | 8 | 0.68 | 0.27-1.71 |  |
| >10 years | 6,159 | 28.0 | 30 | 0.48 | 0.26-0.90 |  |
| OWNED DOGS LEFT FREE TO ROAM |  |  |  |  |  | 0.289 |
| No dogs | 11,963 | 54.3 | 56 | 0.51 | 0.24-1.10 |  |
| No | 6,754 | 30.7 | 30 | 0.44 | 0.22-0.88 |  |
| Yes | 3,310 | 15.0 | 19 | 0.71 | 0.26-1.97 |  |
| OWNED DOGS ALLOWED INTO THE HOUSE |  |  |  |  |  | 0.999 |
| No dogs | 11,963 | 54.3 | 56 | 0.51 | 0.24-1.10 |  |
| No | 9,227 | 41.9 | 46 | 0.51 | 0.23-1.14 |  |
| Yes | 837 | 3.8 | 3 | 0.51 | 0.13-1.93 |  |
| GIVE ANTIPARASITIC TREATMENT TO DOGS |  |  |  |  |  | 0.615 |
| No dogs | 11,963 | 54.3 | 56 | 0.51 | 0.24-1.10 |  |
| Yes, with praziquantel | 2,020 | 9.2 | 10 | 0.44 | 0.21-0.90 |  |
| Yes, with other or unspecified drugs | 1,495 | 6.8 | 5 | 0.30 | 0.08-1.14 |  |
| No | 6,549 | 29.7 | 34 | 0.58 | 0.25-1.37 |  |
| EAT UNWASHED VEGETABLES |  |  |  |  |  | 0.823 |
| No, always washed | 15,389 | 69.9 | 69 | 0.54 | 0.29-1.03 |  |
| Yes, from the market | 235 | 1.1 | 0 | 0.00 | NC |  |
| Yes, from both the market and the garden/fields | 503 | 2.3 | 5 | 0.64 | 0.19-2.12 |  |
| Yes, from the garden/fields | 5,900 | 26.8 | 31 | 0.47 | 0.17-1.28 |  |
| DRINK TAP WATER |  |  |  |  |  | 0.552 |
| No | 6,722 | 30.5 | 30 | 0.43 | 0.24-0.78 |  |
| Yes | 15,305 | 69.5 | 75 | 0.55 | 0.23-1.32 |  |
| DRINK COMMERCIAL WATER |  |  |  |  |  | 0.168 |
| No | 16,466 | 74.8 | 88 | 0.54 | 0.25-1.15 |  |
| Yes | 5,561 | 25.2 | 17 | 0.38 | 0.17-0.85 |  |
| DRINK WATER FROM WELLS |  |  |  |  |  | 0.325 |
| No | 16,281 | 73.9 | 83 | 0.58 | 0.24-1.39 |  |
| Yes | 5,746 | 26.1 | 22 | 0.34 | 0.18-0.66 |  |
| DRINK WATER FROM OTHER SOURCES |  |  |  |  |  | 0.840 |
| No | 21,976 | 99.8 | 105 | 0.51 | 0.24-1.07 |  |
| Yes | 51 | 0.2 | 0 | 0.00 | NC |  |
