# Supplementary material for: Epidemiological factors associated with human cystic echinococcosis: a semi-structured questionnaire from a large population-based ultrasound cross-sectional study in eastern Europe and Turkey
Source: Parasit Vectors. 2019 Jul 29;12:371. doi: 10.1186/s13071-019-3634-1 (PMC6664724; doi:10.1186/s13071-019-3634-1)
Supplement: Supplementary file 3 — Additional file 3. Country specific multivariable analysis. Results of the multilevel logistic regression model, including village as random effect. [file 13071_2019_3634_MOESM3_ESM.docx]

**Additional file 3: Table S2.** Results of the multilevel logistic regression model, including village as random effect. *Accounting for clustering at village level. **Adjusted OR per linear 10-years increase in age. °BULGARIA: variance between villages 0.21 (95% CI 0.02-2.17), intra-village correlation 6.0 (95% CI 0.6-39.7); ROMANIA: variance between villages 0.30 (95% CI 0.03-3.51), intra-village correlation 8.3 (95% CI 0.8-51.6); TURKEY: variance between villages 1.48 (95% CI 0.32-6.96), intra-village correlation 31.1 (95% CI 8.8-67.9).

|  | **BULGARIA°** | | | **ROMANIA°** | | | **TURKEY°** | | |
| --- | --- | --- | --- | --- | --- | --- | --- | --- | --- |
| **Variable** | **Adjusted OR** | **95% CI*** | ***P*-value*** | **Adjusted OR** | **95% CI*** | ***P*-value*** | **Adjusted OR** | **95% CI*** | ***P*-value*** |
| SEX |  |  |  |  |  |  |  |  |  |
| Female | 1 |  |  | 1 |  |  | 1 |  |  |
| Male | 1.21 | 0.66-2.21 | 0.538 | 1.19 | 0.51-2.77 | 0.687 | 0.63 | 0.34-1.16 | 0.138 |
| AGE GROUP** | 0.97 | 0.71-1.33 | 0.837 | 1.19 | 0.87-1.64 | 0.272 | 1.05 | 0.87-1.25 | 0.628 |
| LIVED IN AREAS WITH HIGH DENSITY OF DOGS AND SHEEP  IN THE PAST 20 YEARS |  |  |  |  |  |  |  |  |  |
| No | 1 |  |  | 1 |  |  | 1 |  |  |
| Yes | 2.19 | 0.81-5.96 | 0.124 | NC | - | - | NC | - | - |
| MAIN OCCUPATION IN THE PAST 20 YEARS |  |  |  |  |  |  |  |  |  |
| Non-agricultural activities or office/service employee | 1 |  |  | 1 |  |  | 1 |  |  |
| Housewife | 1.48 | 0.13-16.8 | 0.753 | 1.13 | 0.18-6.99 | 0.894 | 1.92 | 0.93-3.97 | 0.080 |
| Farmer/livestock breeder/other agricultural/veterinary activities | 5.37 | 0.82-35.2 | 0.079 | 2.32 | 0.80-6.66 | 0.119 | 1.08 | 0.31-3.78 | 0.907 |
| Students and children <5 years of age | 3.04 | 1.10-8.32 | **0.032** | 0.42 | 0.03-5.03 | 0.493 | 0.57 | 0.30-1.09 | 0.089 |
| Retired | 5.56 | 0.80-38.7 | 0.083 | 1.43 | 0.34-6.10 | 0.625 | 2.40 | 1.23-4.53 | **0.007** |
| Unemployed | 4.17 | 0.57-30.2 | 0.158 | 0.73 | 0.10-5.31 | 0.757 | NC | - | - |
| AGRICULTURAL ACTIVITIES IN THE PAST 20 YEARS |  |  |  |  |  |  |  |  |  |
| No | 1 |  |  | 1 |  |  | 1 |  |  |
| Yes | 0.74 | 0.23-2.36 | 0.615 | 0.78 | 0.37-1.66 | 0.521 | 2.91 | 1.50-5.67 | **0.002** |
| EDUCATION |  |  |  |  |  |  |  |  |  |
| None | 1 |  |  | 1 |  |  | 1 |  |  |
| Primary | 2.98 | 1.15-7.69 | **0.024** | 0.32 | 0.03-3.11 | 0.330 | 0.95 | 0.38-2.37 | 0.330 |
| Secondary/High school | 1.93 | 0.36-10.2 | 0.440 | 0.35 | 0.04-3.33 | 0.364 | 1.88 | 0.97-3.65 | 0.061 |
| University/Postgraduate | 0.46 | 0.02-9.50 | 0.612 | NC | - | - | NC | - | - |
| KNOWLEDGE OF HUMAN CE EXISTENCE |  |  |  |  |  |  |  |  |  |
| No | 1 |  |  | 1 |  |  | 1 |  |  |
| Yes | 2.08 | 0.38-11.3 | 0.398 | 2.23 | 0.68-7.29 | 0.184 | 1.39 | 0.61-3.16 | 0.427 |
| KNOWN PRESENCE OF RELATIVES WITH CE |  |  |  |  |  |  |  |  |  |
| No | 1 |  |  | 1 |  |  | 1 |  |  |
| Yes | 6.17 | 1.46-26.1 | **0.014** | 1.09 | 0.11-10.8 | 0.942 | 3.92 | 1.45-10.6 | **0.007** |
| RAW VISCERA GIVEN TO DOGS |  |  |  |  |  |  |  |  |  |
| No | 1 |  |  | 1 |  |  | 1 |  |  |
| Yes | 1.37 | 0.58-3.22 | 0.474 | 2.51 | 1.18-5.35 | **0.017** | 1.31 | 0.84-2.03 | 0.232 |
| DRINK COMMERCIAL WATER |  |  |  |  |  |  |  |  |  |
| No | 1 |  |  | 1 |  |  | 1 |  |  |
| Yes | 0.63 | 0.26-1.52 | 0.305 | 0.44 | 0.18-1.08 | 0.074 | 1.01 | 0.50-2.03 | 0.977 |
